# Supplementary material for: Therapeutic microparticles functionalized with biomimetic cardiac stem cell membranes and secretome
Source: Nat Commun. 2017 Jan 3;8:13724. doi: 10.1038/ncomms13724 (PMC5512648; doi:10.1038/ncomms13724)
Supplement: Supplementary Information — Supplementary Figures 1-6 and Supplementary Table 1. [file ncomms13724-s1.pdf]

Supplementary Figure 1

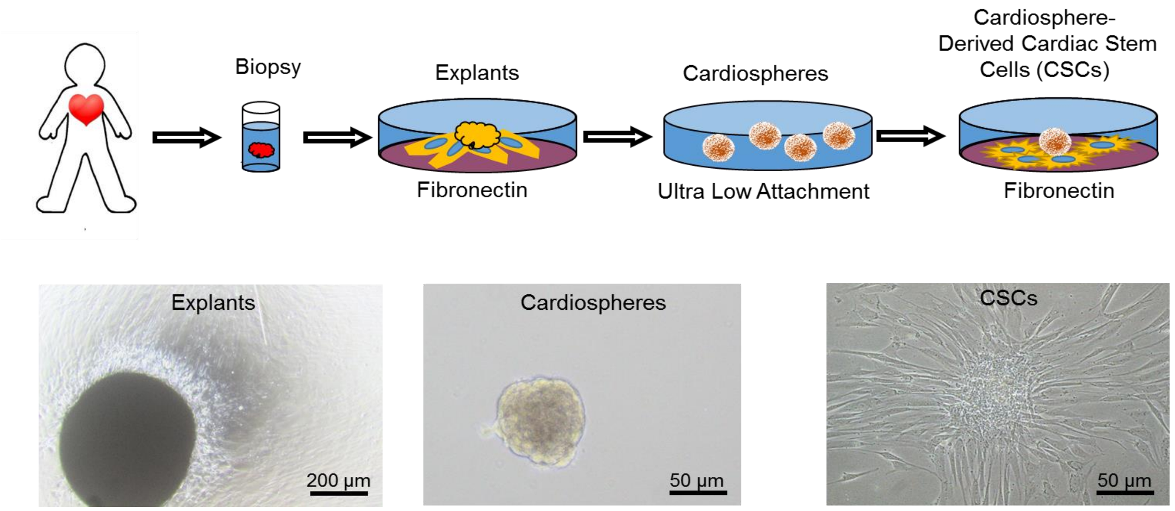

Supplementary Figure 1: Generation and culture of human cardiac stem cells (CSCs). Cardiosphere-derived CSCs were generated with three steps: myocardial tissue explants (scale bar, 200 µm) on fibronectin surface, cardiospheres formed in suspension culture (scale bar, 50 µm), and cardiosphere-derived cardiac stem cells (CSCs) dissociated from cardiospheres on fibronectin-coated surface (scale bar, 50 µm).

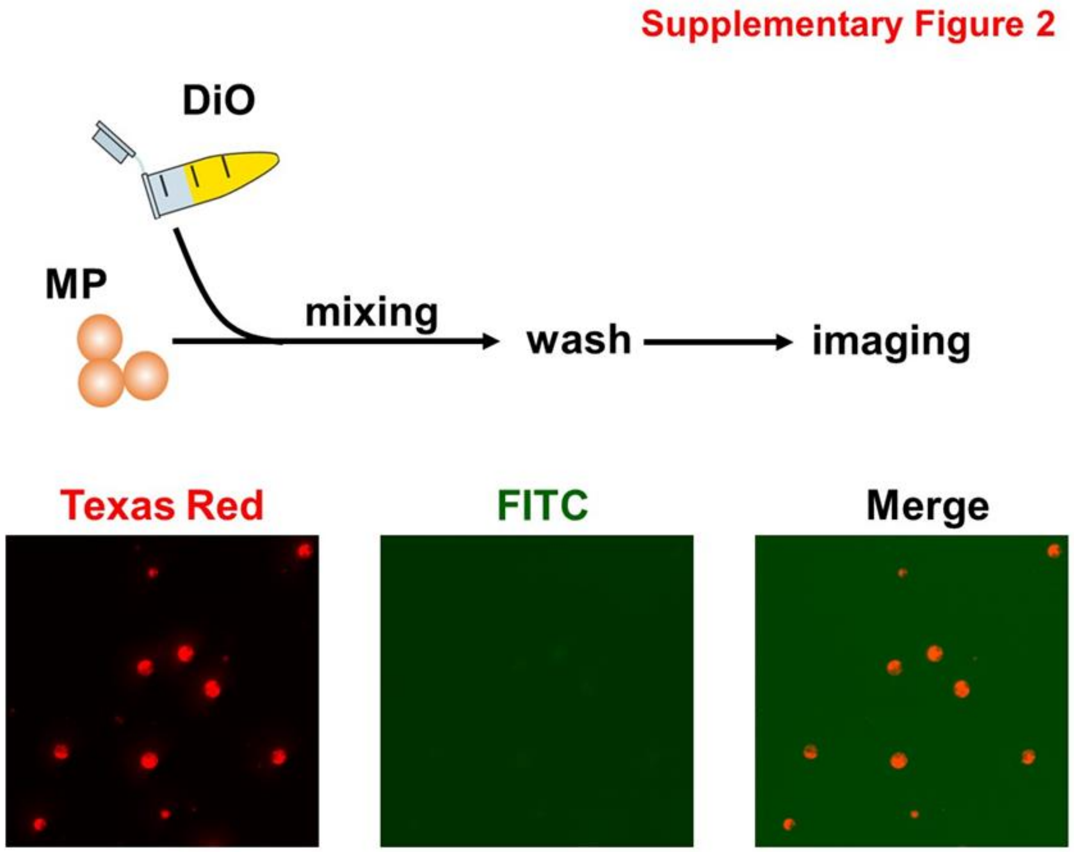

Supplementary Figure 2: Rule out of nonspecific DiO binding to microparticles. Schematic showing experimental design. DiO was incubated with Texas red succinimidyl ester-labeled labeled Control-MP_1 for 30 mins following three times wash by PBS.

Supplementary Figure 3

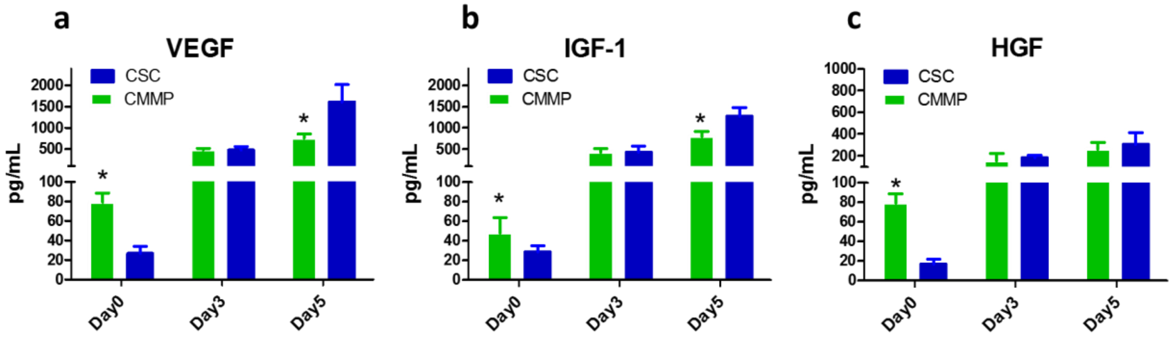

Supplementary Figure 3: Comparison of regenerative factors released by cell-mimicking microparticles (CMMPs) and cardiac stem cells (CSCs). Release of vascular endothelial growth factor (VEGF) (a), insulin-like growth factor (IGF)-1 (b) and hepatocyte growth factor (HGF) (c) from CMMPs (green bar) or CSCs (blue bar) at various time points determined by ELISA. n = 3 for each group at each time points. All data are mean ± s.d. Comparisons between any two groups were performed using two-tailed unpaired Student's *t*-test. * indicated P<0.05 when compared to CSC group.

Supplementary Figure 4

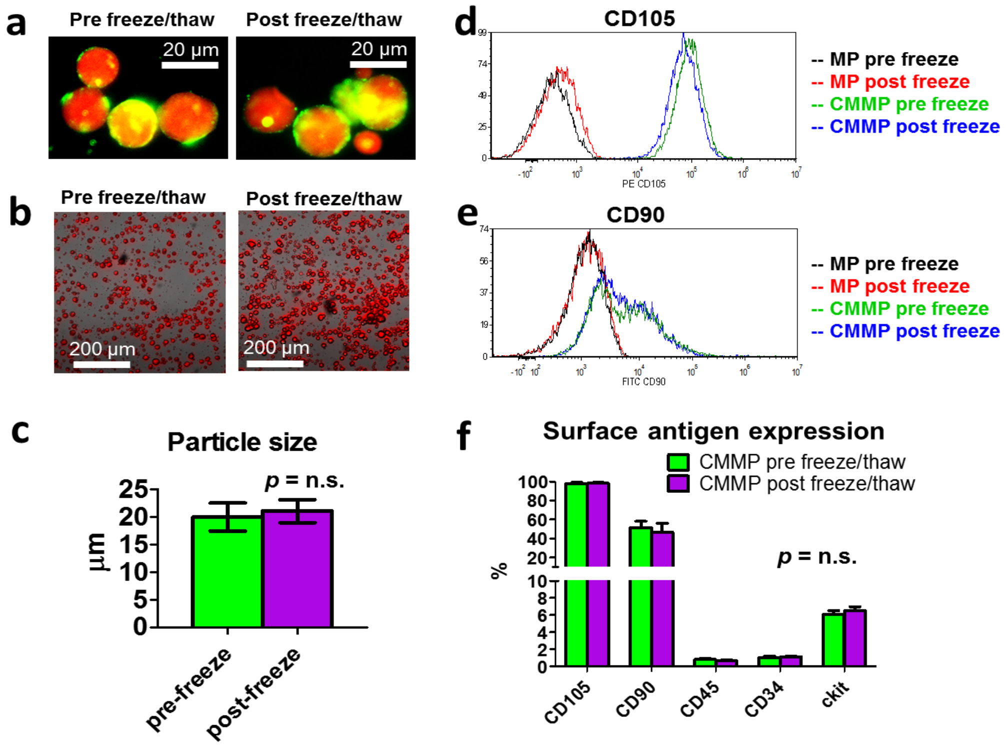

Supplementary Figure 4: Physiochemical and biological properties of cell-mimicking microparticles (CMMPs) pre- and post-freeze/thaw. (a) Representative fluorescent images of CMMPs snap frozen at -80 °C and thawed in water. Scale bar, 20 µm. (b, c) Representative images of CMMPs (b) and quantitation of particle size pre- and post-freeze/thaw. Scale bar, 200 µm. n = 3 for each group. NS indicated *P*>0.05. (d, e) Freezing and thawing did not alter the expressions of CD105 (d) and CD90 (e) on CMMPs. (f) Surface antigen expressions on CMMPs were not affected by freezing and thawing. n = 3 for each group. NS indicated *P*>0.05. All data are mean ± s.d. Comparisons between any two groups were performed using two-tailed unpaired Student's *t*-test.

Supplementary Figure 5

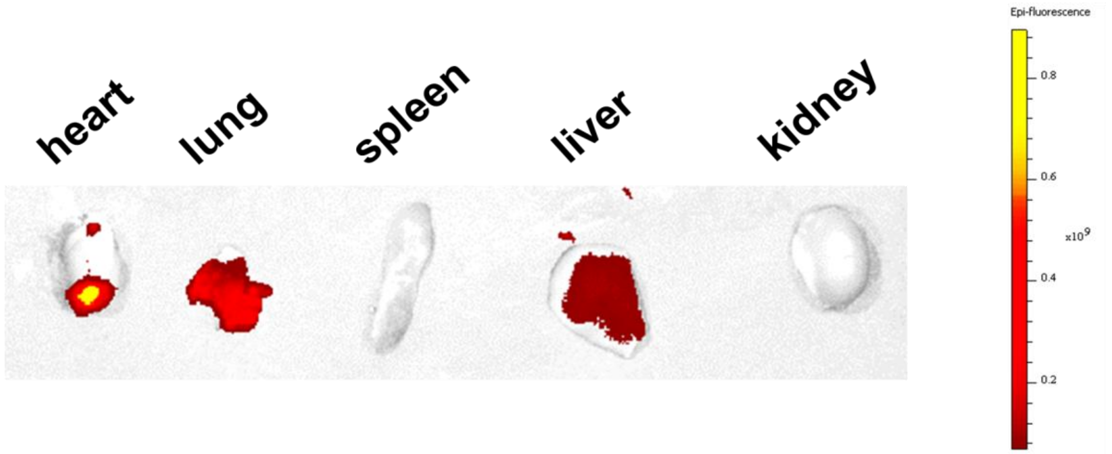

Supplementary Figure 5: Biodistribution of cell-mimicking microparticles (CMMPs) 7 days after injection. Representative *ex vivo* fluorescent imaging indicated that the majority of CMMPs remained in the heart after injection, while "washed away" CMMP signal could be found in the lung and the liver.

Supplementary Figure 6

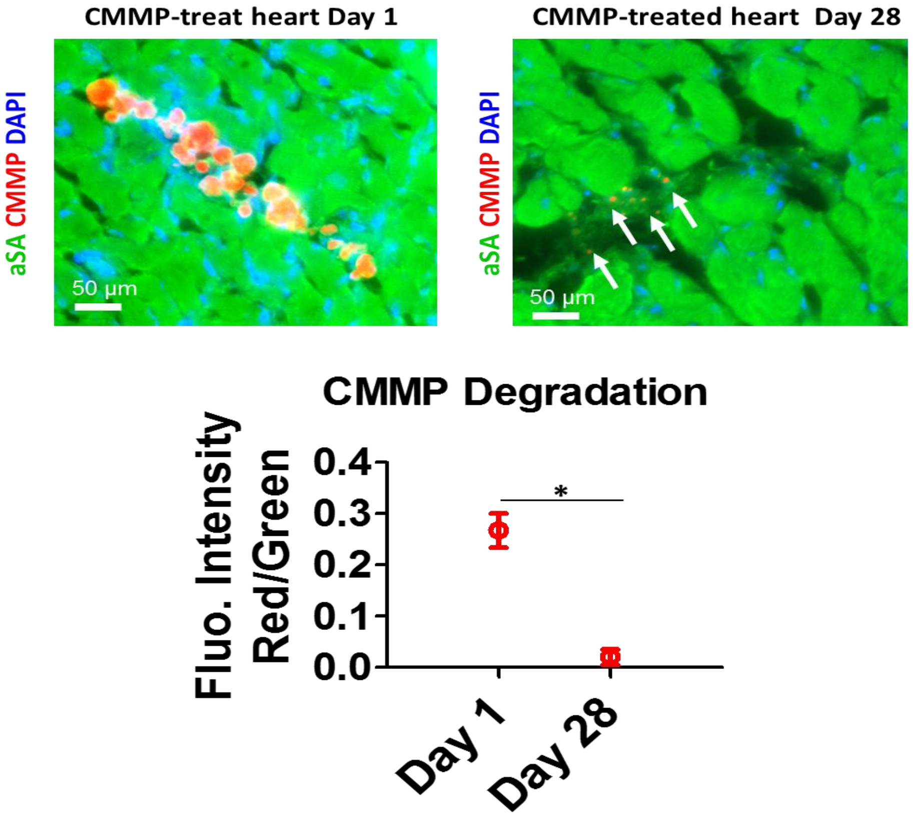

Supplementary Figure 6: Degradation of cell-mimicking microparticles (CMMPs) *in vivo.* Representative microscopic images showing injected CMMPs (red) in post MI hearts at Day 1 and Day 28. White arrow indicated the trace amount of CMMPs at Day 28. Scale bar, 50 μm. Quantitation of CMMPs degradation as reflected by the decrease of red fluorescence. n = 3 animals per group. * indicated $P<0.05$ when compared to “Day 1” values. All data are mean ± s.d. Comparisons between any two groups were performed using two-tailed unpaired Student’s *t*-test.

Supplementary Table 1

Characteristics	Cardiac Stem Cells	Cell-mimicking microparticles (CMMPs)
Secretion of regenerative factors	Yes	Yes
Binding to host cells	Yes	Yes
Ability to differentiate into host cells	Yes (but very rare)	No
Ability to proliferate	Yes (limited proliferation *in vivo*)	No
Ability to leave blood vessels after delivery	Yes	Yes
Therapeutic benefits in myocardial infarction	Yes	Yes
Cryopreservation and storage without affecting quality	No	Yes
Stimulate local T cell immune response	Yes	No

Supplementary Table 1: Summarized comparison between cardiac stem cells and CMMPs.
